# The cost of unresectable stage III or stage IV melanoma in Italy

**DOI:** 10.1186/1756-9966-31-91

**Published:** 2012-11-01

**Authors:** Michele Maio, Paolo Ascierto, Alessandro Testori, Ruggero Ridolfi, Emilio Bajetta, Paola Queirolo, Michele Guida, Antonella Romanini, Vanna Chiarion-Sileni, Jacopo Pigozzo, Anna Maria Di Giacomo, Mario Calandriello, Guido Didoni, Marck van Baardewijk, Cyril Konto, Carlo Lucioni

**Affiliations:** 1 Medical Oncology and Immunotherapy, Azienda Ospedaliera Universitaria Senese, Istituto Toscano Tumori, Strada delle Scotte, 53100, Siena, Italy; 2 Istituto Nazionale Tumori, Fondazione Pascale, Naples, Italy; 3 Istituto Europeo di Oncologia, Milan, Italy; 4 IRCCS - IRST, Meldola, Forlì-Cesena, Italy; 5 Istituto di Oncologia, Policlinico di Monza, Italy; 6 Oncologia Medica A, Azienda Ospedaliera S. Martino-, Istituto Nazionale per la Ricerca sul Cancro IST, Genoa, Italy; 7 Istituto Tumori Giovanni Paolo II, IRCCS, Bari, Italy; 8 Oncologia Medica 1, AOU Pisana “Spedali Riuniti di Santa Chiara”, Pisa, Italy; 9 Istituto Oncologico Veneto, IRCCS, Padova, Italy; 10 Health Economics & Outcome Research Unit, BMS Italy, Rome, Italy; 11 Head Market Access & External Affairs, Novartis Oncology, Amsterdam, Netherlands; 12 Global Clinical Research Oncology, BMS Global, Wallingford, Oxfordshire, UK; 13 Health Economist, Springer Healthcare, Milan, Italy

**Keywords:** Metastatic melanoma, Medical direct cost, Clinical outcomes

## Abstract

**Background:**

In recent decades, melanoma incidence has been increasing in European countries; in 2006, there were approximately 60,000 cases leading to 13,000 deaths. Within Europe there is some geographical variation in the incidence of melanoma, with the highest rates reported in Scandinavia (15 cases per 100,000 inhabitants per year) and the lowest in the Mediterranean countries (5 to 7 cases per 100,000 inhabitants per year).

**Methods:**

The present article is based on the information collected in the MELODY study (MELanoma treatment patterns and Outcomes among patients with unresectable stage III or stage IV Disease: a retrospective longitudinal survey).

In that study, the medical charts of patients were reviewed to document current treatment patterns and to analyse information on patients, disease characteristics and healthcare resource utilization related to the treatment of advanced melanoma regarding patients who presented with a diagnosis of malignant melanoma (stage I to IV) at participating sites between 01 July, 2005 and 30 June, 2006.

**Results:**

Summarizing, though the length of the follow-up period varies among sample patients, an amount of the yearly cost per patient can be estimated, dividing the average per patient total cost (€ 5.040) by the average follow-up duration (17.5 months) and reporting to one year; on these grounds, unresectable stage III or stage IV melanoma in Italy would cost € 3,456 per patient per year.

## Background

In recent decades, melanoma incidence has been increasing in European countries; in 2006, there were approximately 60,000 cases leading to 13,000 deaths [1,2].

Within Europe there is some geographical variation in the incidence of melanoma, with the highest rates reported in Scandinavia (15 cases per 100,000 inhabitants per year) and the lowest in the Mediterranean countries (5 to 7 cases per 100,000 inhabitants per year) [3,4].

Risk factors for melanoma include family history of the disease, presence of multiple moles and a previous melanoma [5]. Epidemiological studies have shown acute and intermittent sunlight exposure is a major environmental etiological factor of malignant melanoma, but the evidence for the causative role of sunlight is still conflicting. Physical protection from exposure to sunlight is generally accepted as the most important factor of melanoma risk reduction. Active public education campaigns aimed at encouraging earlier detection of melanoma have led to the diagnosis of thinner lesions with a better prognosis [3,6].

Although melanoma accounts for only 4 percent of all skin cancers, it is responsible for 80 percent of deaths from this type of cancer and causes disproportionate mortality in patients of young and middle age [5,6]. Estimates of mortality rate from melanoma in Europe vary between 1.5 to 5.2 per 100,000 inhabitants per year [1].

More recent improvements in survival have been attributed in part to the earlier detection of melanoma. If the disease becomes metastatic, it is considered incurable. The prognosis for patients with distant metastasis remains bleak, with an estimated median survival of 6 to 10 months and less than 5 percent of patients surviving for more than 5 years [7].

Local recurrences of malignant melanoma and in-transit metastasis are most effectively treated by surgical excision. Radiotherapy to bone or skin metastases can provide short term symptomatic control and offer palliative value, but patients in Europe with unresectable metastatic disease have very few systemic treatment options. Dacarbazine, an alkylating agent, is approved in Europe for the treatment of metastatic melanoma [6,8]. A number of other agents, including temozolomide and fotemustine, have been investigated for treatment of metastatic melanoma and because of their ability to cross the blood–brain barrier, may be used preferentially in melanoma patients with brain metastasis. However, no agent has been shown to improve survival rates. Immunotherapy with interleukin-2, approved by the FDA in the United States, did not receive approval for the treatment of metastatic melanoma in Europe. Little progress has been made in the medical treatment of metastatic melanoma in the last 3 decades [9].

The limited number of approved treatments for advanced melanoma patients suggests there is a high, unmet medical need for new therapies [10,11].

## Methods

In the development of new treatments, it is important to have an understanding of existing treatment options. In diseases such as advanced melanoma where few approved and effective treatment options exist, clinicians may adopt different approaches to manage patients’ disease. Documenting and characterizing current treatments and their associated cost is important to define the dominant treatment practice and to quantify the impact of existing therapeutic strategies in terms of both clinical benefit for the patient, as well as cost to the healthcare system. Consequently the primary objective of this study is to document treatment patterns and evaluate relevant costs. In particular, to document first-line, second-line and beyond treatments types as well as the frequency with which they are used in patients diagnosed with unresectable stage III or stage IV melanoma.

The present article is based on the information collected in the MELODY study (MELanoma treatment patterns and Outcomes among patients with unresectable stage III or stage IV Disease: a retrospective longitudinal surveY).

In that study, the medical charts of patients were reviewed to document current treatment patterns and to analyse information on patients, disease characteristics and healthcare resource utilization related to the treatment of advanced melanoma. Moreover, the perspective of the Italian National Health System is adopted, so only direct costs are considered.

### The MELODY study

The MELODY study was conducted as a multinational, observational retrospective longitudinal survey of patients diagnosed with unresectable stage III or stage IV melanoma. The target sample population was approximately 750 patients from 3 European countries: France, Italy and the United Kingdom. In each country 10 sites were selected, providing approximately 250 patients per country. In each participating site, consecutive patients with a diagnosis of malignant melanoma (stage III to IV) who presented at the site between 01 July 2005 and 30 June 2006 were entered into a registry where a limited set of parameters related to date and stage of disease was captured. Staging was in accordance with the American Joint Commission on Cancer (AJCC 2001) criteria [12]. Each site entered patients into the registry up to a maximum of 250 patients or until 25 eligible patients (those with a diagnosis of unresectable stage III or stage IV melanoma) were identified (whichever occurred first).

For each patient who met all inclusion criteria, medical chart data were abstracted beginning from the date of unresectable stage III or stage IV diagnosis until 01 May, 2008 or death, whichever occurred first. Given an estimated median survival of 6 to 10 months in the patient population, the duration of the follow-up from diagnosis until 01 May 2008 allowed an adequate time to collect information on treatments received, patient and disease characteristics, and health resource utilization. The patient identity (name, address and other identifiers) was not collected and ethics committee approval and patient informed consent were obtained.

Treatment data were collected by line of therapy. Data included systemic therapy (chemotherapy, immunotherapy), surgery, radiation, supportive care only, enrolment in a clinical trial or no treatment. For systemic therapy, name of the drug, schedule and method of administration, duration of treatment and reason for stopping treatment were collected. If a patient was enrolled in a clinical trial for treatment of advanced melanoma, the duration of the participation in the trial was noted in the case report form, but no further details (name of drug, schedule of administration) were collected.

### Healthcare resource utilization

Categories of healthcare resource utilization included hospitalizations, outpatient visits, emergency department visits, hospice care, surgery, radiotherapy and management of adverse events (transfusions and concomitant medications including antiemetics and growth factors) related to the treatment of unresectable stage III or stage IV melanoma. Resource use related to treatments received as a part of a clinical trial was not reported.

In the MELODY study data were also collected on clinical benefits and outcomes of the treatments (response rate, disease control rate, time to response, duration of response and progression free survival). In this article only the response rate has been considered, in order to evaluate the level of costs per patient respectively responsive and non responsive to systemic therapy, stratifying by line and type of treatment.

Due to the anticipated small proportion of patients who receive second-line treatment and beyond, later lines of treatment were not characterized with the same level of precision as first-line treatments, hence caution should be applied in the interpretation of the findings regarding those lines. Loss to follow-up (including patients who stop treatment prematurely, transfer out of the treatment facility or death not documented in the patient’s medical chart) is an inherent limitation of any retrospective study design. However, due to the short median duration of survival and to the frequent contacts between clinicians treating patients with advanced disease, loss to follow-up was low. For this reason, without compromising the sample size, only patients having a follow-up of ≥ 2 months were included in the study, in order to minimize the number of patients whose melanoma was not treated or for whom no information on treatment was available.

### Database methodology and statistical analysis

Patient and disease characteristics include patient age, gender, date and disease stage at first melanoma diagnosis, date and disease stage at advanced (stage III unresectable or stage IV) melanoma diagnosis (according to AJCC 2001 criteria) [12].

For each line of treatment (excluding treatments received as a part of a clinical trial), the number and duration of hospitalizations, the duration of hospice care, the number of outpatient visits and the number of emergency room visits related to the treatment of unresectable stage III or stage IV melanoma were recorded. Resource use associated with common adverse events (transfusion, administration of concomitant medications including anti-emetics and growth factors) was recorded too.

Statistical analyses are predominantly descriptive in nature, presented as summary tables and including calculation of measures of central tendency and standard deviations for continuous variables and frequency distributions for categorical variables. The following analyses were performed on the sample data relative to the Italian patients.

### MELODY study: the Italian sub-study

#### Stratification variables

**Figure 1:**
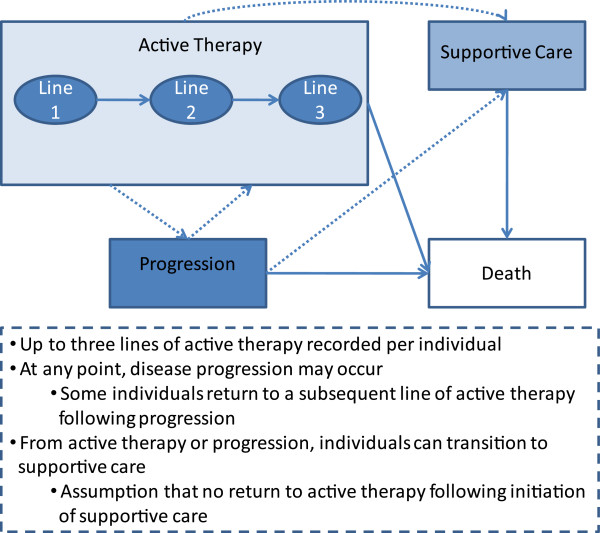
Summary of potential patient pathways through treatment and health states in the MELODY study.

The population of interest included all patients in the participating Italian sites diagnosed with unresectable stage III or stage IV melanoma who received active treatment with systemic therapy, outside of a clinical trial, and/or any form of supportive care. Inclusion in this population varied across therapy lines, as shown in Figure 1. Up to three lines of active therapy were recorded per patient but, at any point of the treatment, disease progression might occur and some patients return to a subsequent line of active therapy following progression. From active therapy or progression, patients might move to supportive care, with the assumption of no return to active therapy following start of supportive care.

Within each line of therapy, all resource utilization variables were recorded for eligible patients receiving systemic therapy. Surgeries and radiotherapy were also recorded for patients receiving these therapies in combination with systemic therapy. If a patient switched from active therapy to supportive care, a subset of resource utilization variables were recorded (hospitalization, outpatient, emergency room, hospice care).

Within each line of active therapy, response was classified into five levels: complete response, partial response, stable disease, no response, and unable to determine. For the cost analyses at the therapy line level, different response status were grouped into two levels: any response (complete, partial, or stable disease) vs. no documented response (no response or unable to determine). For the cost analysis at the overall level, patients were classified as having any response if they had a documented response to any line of therapy, vs. no response if they did not have a documented response to any line of therapy.

Patient follow-up time was reported and used in calculating outcomes per unit time. Follow-up time was considered both overall and within lines of treatment and was calculated as follows:

- Overall follow-up time was defined as the length of time between first date of active therapy and last active date, where last active date is defined to be the date of last contact, death date, or censor date as appropriate for each patient.

- Follow-up time on a line of active therapy was defined as the difference between start date of the therapy and start date of next therapy for patients who went on to receive further active therapy or supportive care, or the difference between therapy start date and last contact date for patients who did not receive any further therapy.

#### Sample profile

The total number of patients was stratified in three lines of active therapy plus supportive care. At the end of the follow-up, the same patient might have been included in more than one line of therapy (due to successively moving from one to another).

#### Outcome variables stratification

All outcomes relating to intensity of resource utilization were stratified by line of therapy and by response rate. Due to low outcome rates, for hospice care, emergency room visits and transfusion, no stratification was considered. For adverse events the only stratification considered was per line of therapy, as response status is not of interest with respect to adverse events. Medication use was adopted as a proxy for adverse events incidence and duration.

### Italian unit costs

Table 1 shows unit costs for Italy in 2009 euro values. Unit costs were obtained from several sources (when available, from published microcosting analysis or from published articles). When real costs were not available, current tariffs (mainly DRG ones) were used as a proxy. The costs of medical management agents for adverse events were calculated using an algorithm where adverse events were classified into categories based on ATC (Anatomical Therapeutic Chemical - level 2) of the drugs used for their treatment. Daily drug cost was based on most frequently prescribed medication. When necessary, original cost data were inflated to 2009 via consumer price index. More detailed information on unit cost can be found on notes included in Table 1, and in relevant references there quoted.

**Table 1:** Treatment of advanced melanoma in Italy - Unit costs

**Resource use item**	**Unit**	**Cost (€ 2009)**	**Notes**	**Source**
Hospitalization	cost per day	740	Cost for one day stay in hospital, overall average. Original data referred to 2004, inflated to 2009 via consumer price index	[13]
Hospice stay	cost per day	211	Daily current tariff, mean of Lombardy and Piedmont values	[14]
Emergency room visit	cost per visit	252	Original cost data referred to 2007, inflated to 2009 via consumer price index	[15]
Outpatient (specialist visit)	cost per visit	22	Specialist visit, current tariff (code: 89.7)	[16]
Adverse events (AE)	cost per day	see Note	AEs classified into categories based on ATC coding (level 2) of the drugs used for their treatment. Daily drug cost based on most frequently prescribed medications (e.g. ondansetron, filgrastim, lenograstim, pegfilgrastim, etc.)	[17]
Radiotherapy	cost per regimen in combination with systemic therapy	2814	DRG 409 (radiotherapy in day hospital) current tariff times average radiotherapies/patient number (7.5)	[18,19]
Transfusion	cost per procedure	179	Current tariff for one unit (ml 280 +/− 20%) of red blood cells added to transfusion procedure tariff (code: 99.07.1)	[16,20]
SURGERY				
Resection of primary tumor	cost per procedure	2785	DRG 266 tariff	
Lymph node resection	cost per procedure	1359	DRG 270 tariff	[18]
All other visceral	cost per procedure	7322	Average of DRG tariffs (192: liver and pancreas; 149: abdomen; 303: kidney)	[18]
Brain metastases	cost per procedure	13493	DRG 001 tariff	[18]
Isolated limb perfusion	cost per procedure	2411	DRG 273 tariff	[18]
Biopsy	cost per procedure	14	Procedure tariff (code: 86.11)	[16]
Distant skin	cost per procedure	2072	Average of DRG 266 and 270 tariffs	[18]
Lung	cost per procedure	8335	DRG 75 tariff	[18]

## Results

### Characteristics of the study sample

**Figure 2:**
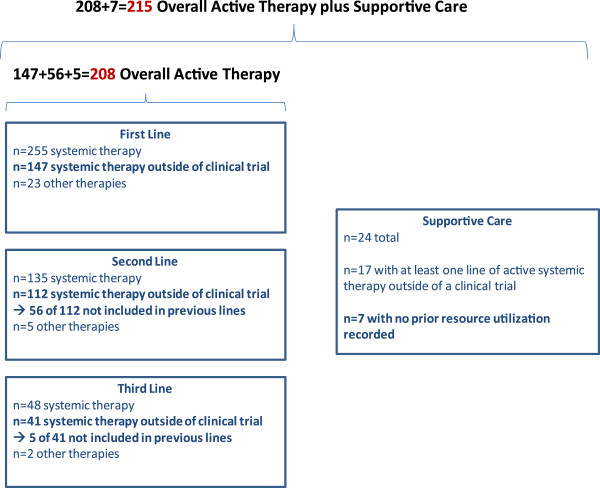
Sample sizes of individuals eligible to contribute resource utilization data during active therapy and supportive care in Italy.

Table 2 reports descriptive statistics of the sub-study sample. The sample included 215 patients, who were eligible to contribute resource utilization data having received active therapy only (191), active therapy and supportive care (17) and supportive care without prior resource utilization (7). Moreover, 147 received first- line therapy, 112 second-line therapy and 41 third-line therapy (Figure 2). Stratification per line of active therapy considered 300 therapeutic treatments, a larger number than the total of patients receiving active therapy (208), because the same patient might have received more than one line of therapy.

**Table 2:** Descriptive statistics of study sample

	**Italy population (N = 215)**	
**Age at diagnosis (years)**	**Mean**	**SD**
	55	13,9
Sex	N	%
Male	135	62,9%
Female	80	37,1%
Total receiving active systemic therapy	N	%
Overall	208	96,7%
First-line	147	68,4%
Second-line	112	52,1%
Third-line	41	19,1%
of which:		
any response to systematic therapy	N	%
Overall	89	42,8%
First-line	53	36,1%
Second-line	34	30,4%
Third-line	14	34,1%
Total follow-up time (months)	Mean	SD
Overall	17,5	13
First-line	9,9	10,7
Second-line	8,9	7,7
Third-line	7,7	6,7
Supportive care	4,9	5
Received supportive care	N	%
Total	24	11,2%
with at least one line of active systematic therapy	17	7,9%

The different major therapy options used are: 33% first line patients were treated with dacarbazine, 20% with fotemustine, and 12% with a combination of dacarbazine+fotemustine; in second line, 51% of patients were treated with fotemustine, and 10% with dacarbazine; in third line, fotemustine was used for 40% of patients, while dacarbazine for 8% of patients.

The mean age at the diagnosis was 55 years and male patients represented 62.9% of the sample. Among the 300 therapeutic treatments 42.8% showed some response to systemic therapy. Within each line of therapy – that is net of double counting – response rate was lower (36.1% in the first line, 30.4% in the second line and 34.1 in the third line).

The total length of follow-up time was 17.5 months, with lower durations in the first line (9.9 months) in the second line (8.9 months) and in the third line (4.9 months).

### Hospitalization

Hospitalizations were not particularly frequent, with less than 10% of all patients experiencing it. Hospitalization tended to be more frequent (12.4% vs 5.9%) for patients with any response to systemic therapy in comparison with those with no response (Table 3, Table 4 and Table 5). Hospitalization was the most expensive category of resource utilisation, both among those who experienced hospitalization (mean total cost of € 25,540) and with reference to the generality of the sample (i.e. including patients with zero utilisation): € 2,481. Moreover, the mean cost per patient with any response to systemic therapy was higher than the mean cost per patient with no response (€ 4,524 vs € 882); the mean cost per patient in the first line of therapy (€ 2,634) was higher than the overall cost (€ 2,481), and much higher than the mean cost per patient in the second (€ 588) and third (€1.356) line of therapy.

**Table 3:** Summary statistics for hospitalizations for patients receiving systemic therapy and/or supportive care

		**Overall**	**First-line therapy**	**Second-line therapy**	**Third-line therapy**	**Supportive care**
N		215	147	112	41	24
Patients with any hospitalization	N	21	11	7	4	4
	%	9,8%	7,5%	6,3%	9,8%	16,7%
Total length of hospitalization (days)	Mean	34,3	47,5	12,7	18,8	8,2
	95%CI	0-73,7	0-126,6	6,6-18,8	0-38,9	1,1-15,4
Length of hospitalization (days/month^(1)^)	Mean	1,9	11,6	6,1	7,5	19,8
	95%CI	0,6-3,2	0-30,8	0-15,3	0-27,4	0-74,2
Total hospitalization cost per hospitalized patient (€ 2009)	Mean	25.400	35.200	9.400	13.900	6.100
	95% CI	0-54.500	0-93.700	4.900-13.900	0-28.800	800-11.400
Total hospitalization cost per hospitalized patient per month (€ 2009)	Mean	1.400	8.600	4.500	5.600	14.700
	95% CI	400-2.400	0-22.800	0-11.300	0-20.300	0-54.900
Total hospitalization cost per patient (€ 2009)	Mean	2.481	2.634	588	1.356	1.017

^(1)^ month of follow-up.

**Table 4:** Summary statistics for hospitalizations for patients with any response to systemic therapy

		**Overall**	**First-line therapy**	**Second-line therapy**	**Third-line therapy**
N		89	53	34	14
Patients with any hospitalization	N	11	5	4	1
	%	12,4%	9,4%	11,8%	7,1%
Total length of hospitalization (days)	Mean	49,5	13,4	10,5	16
	95% CI	0-129,9	2,2-24,6	0,6-20,4	NA
Length of hospitalization (days/month^(1)^)	Mean	3	3,3	1,2	1,4
	95%CI	1,4-4,6	0,9-5,8	0,6-1,8	1,4-1,4
Total hospitalization cost per hospitalized patient (€ 2009)	Mean	36.600	9.900	7.770	11.800
	95% CI	0-96.100	1.600-18.200	400-15.100	NA
Total hospitalization cost per hospitalized patient per month (€ 2009)	Mean	2.200	2.400	888	1.000
	95% CI	1.000-3.400	700-4.300	400-1.300	1.000-1.000
Total hospitalization cost per patient (€ 2009)	Mean	4.524	934	914	843

^(1)^ month of follow-up.

**Table 5:** Summary statistics for hospitalizations for patients with no response to systemic therapy

		**Overall**	**First-line therapy**	**Second-line therapy**	**Third-line therapy**
N		119	94	78	27
Patients with any hospitalization	N	7	6	3	3
	%	5,9%	6,4%	3,8%	11,1%
Total length of hospitalization (days)	Mean	20,3	76	15,7	19,7
	95% CI	10,5-30,1	0-243,6	0-33,3	0-57,7
Length of hospitalization (days/month^(1)^)	Mean	2,5	18	10,9	7,4
	95%CI	1,9-3	0-39,3	2-19,8	0-17,1
Total hospitalization cost per hospitalized patient (€ 2009)	Mean	15.000	56.200	11.600	14.600
	95% CI	7.800-22.300	0-180.300	0-24.600	0-42.700
Total hospitalization cost per hospitalized patient per month (€ 2009)	Mean	1.900	13.300	8.100	5.500
	95% CI	1.400-2.200	0-29.100	1.500-14.700	0-12.700
Total hospitalization cost per patient (€ 2009)	Mean	882	3.587	446	1.622

^(1)^ month of follow-up.

As previously pointed out, the same patient might be included in more than one sub-set (first-line, second-line and third-line). But this event raises perplexities when making comparisons, with discrepancies that are particularly evident when results are analysed separately for patients with any response to systemic therapy and with no response to systemic therapy (Tables 4 and Table 5). For example, the overall mean length of stay does not correspond to the mean of the line-specific length of stay in Table 4 (“Any response to therapy”). This is due to the definition of responders. Within each line of therapy, patients are classified as responders or non-responders, and their results are included in the corresponding table. For the Overall column, a patient is included in the “Any Response” table if he/she did ever respond to a single line of therapy, and is included in the “No Response” table if he/she never did. So, considering a hypothetical patient who responded to first-line therapy but not to second-line or third-line therapy, he would be included in the Overall column and in the first-line column in Table 4 (since he did respond to this line of therapy); and he would be included in the second- and third-line columns in Table 5 (because he did not respond to these lines of therapy). Consequently Overall columns in Table 4 (and analogously in Table 5) do not correspond to an average of the line-specific columns because patients can move across tables^a^.

These methodological considerations are done here to justify why results will not be commented separately per single line of treatment, when patients are analyzed with any/no response to systemic therapy.

Summarizing, though the length of the follow-up period varies among sample patients, an amount of the yearly cost per patient can be estimated, dividing the average per patient total cost (€ 5.040) by the average follow-up duration (17.5 months) and reporting to one year; on these grounds, unresectable stage III or stage IV melanoma in Italy would cost € 3,456 per patient per year.

### Hospice care

Approximately 6% of patients received hospice care with a mean cost per admitted patient of € 3.300. Due to the low frequency of such resource use, the mean cost for the generality of the sample is quite low (€ 184).

### Emergency room visit

Emergency room visits were very rare: overall 1.4% of patients had one or more visit. Consequently the mean cost for the generality of the sample is very low (€ 4).

### Outpatient visit

Outpatient visits were the most common category of resource utilization: 40.5% of patients had at least one visit, with 3.3 visits per patient (Overall) on average. As compared with other major categories of utilization, outpatient visits were relatively inexpensive, with a mean cost of € 70 per visited patient and a mean cost for the generality of the sample of € 28 (Table 6). Outpatient visits were more frequent in patients with any response to systemic therapy, where the mean cost per patient was higher than the mean cost per non responder patient (€ 33 vs € 22).

**Table 6:** Summary statistics for outpatient visits for patients receiving systemic therapy and/or supportive care

		**Overall**	**First-line therapy**	**Second-line therapy**	**Third-line therapy**	**Supportive care**
N		215	147	112	41	24
Patients with any outpatient visits	N	87	44	36	19	15
	%	40,5%	29,9%	32,1%	46,3%	62,5%
Total number of outpatient visits per visited patient	Mean	3,3	2,4	2,5	2,5	2,7
	95%CI	2,8-3,7	2,1-2,8	2-3	1,8-3,2	1,9-3,4
Total number of outpatient visits per visited patient per month ^(1)^	Mean	0,3	0,5	0,6	0,5	3,3
	95%CI	0,2-0,4	0,3-0,7	0,3-0,9	0,4-0,7	0-7,1
Total outpatient cost per visited patient (€ 2009)	Mean	70	50	60	50	60
	95% CI	60-80	50-60	40-70	40-70	40-80
Total outpatient cost per visited patient per month (€ 2009)	Mean	7	11	13	11	73
	95% CI	4-9	7-15	7-20	9-15	0-156
Total outpatient cost per patient (€ 2009)	Mean	28	15	19	23	38

^(1)^ month of follow-up.

### Adverse events

On the whole, 24% of patients received medications to manage one or more adverse events (AE). Most of the patients experienced just one AE requiring medical management. The most frequent category of AE medical management agent was antiemetics and antinauseants, the most expensive category of medication was immunostimulants, ranging from € 785 to € 3,051 per episode (Table 7).

**Table 7:** Cost of adverse event management for most commonly prescribed agents (occurring in ≥ 5% of patients

**Category of adverse event management**	**Most frequent medical agent(s)**	**Percentage of events treated with agent**	**Unit cost per day (€ 2009)**	**Mean duration (days)**	**Cost per event (€ 2009)**
Antiemetics and antinauseants	Ondansetron ^(1), (2)^	90,7	5,99	66,5	56,9
Drugs for acid related disorders	Omeprazole	75	0,25	99,5	24,9
Corticosteroids for systemic use	Dexamethasone	50	0,8	133,3	106,6
Analgesics	Co-efferalgan	30,8	0,52	48,5	25,2
	Tramadol	30,8	1,92	25,5	49
Drugs for functional gastrointestinal disorders	Metoclopramide	100	0,92	97,5	89,7
Immunostimulants	Filgrastim ^(3)^	44,4	65,42	23,2	785
	Lenograstim	11,1	79,39	12	952,7
	Pegfilgrastim ^(4)^	11,1	902,48	71	3051,2

^(1)^ Assumed maximum duration 3 days per 21-day cycle throughout observed mean duration.

^(2)^ Unit cost is per day, given once per 21-day cycle throughout observed mean duration.

^(3)^ Assumed maximum duration 12 days; if observed mean duration of 23,2 days is used, then total cost is 1517,7.

^(4)^ Unit cost is per cycle, given once per 21-day cycle throughout observed mean duration.

### Radiotherapy

Among patients who received systemic therapy, 19.7% received radiotherapy in combination (Tables 8 and 9). Radiotherapy costs were based on standard protocols regimens. Mean cost per patient receiving radiotherapy was equal to the unit cost of this resource (€ 2.814). Mean cost per patient for the generality of the sample resulted equal to € 555. Small differences in mean cost per patient with any response (€ 506) vs no response (€591) are due to the different frequency in the resource use (18.05% vs 21%).

**Table 8:** Summary statistics for radiotherapy for patients receiving systemic therapy

		**Overall**	**First-line therapy**	**Second-line therapy**	**Third-line therapy**
N		208	147	112	41
Patients with any radiotherapy	N	41	24	13	6
	%	19,7%	16,3%	11,6%	14,6%
Incidence of radiotherapy (per patient with any radiotherapy per month ^(1)^)	Mean	0,1	0,31	0,27	0,14
	95% CI	0,08-0,13	0,13-0,5	0,07-0,46	0,03-0,24
Total radiotherapy cost per patient with any radiotherapy (€ 2009)	Mean	2.814	2.814	2.814	2.814
Total radiotherapy cost per patient with any radiotherapy per month (€ 2009)	Mean	300	900	800	400
	95% CI	200-400	400-1.400	200-1.300	100-700
Total radiotherapy cost per patient (€ 2009)	Mean	555	459	327	412

^(1)^ month of follow-up.

**Table 9:** Summary statistics for radiotherapy for patients with any response to systemic therapy

		**Overall**	**First-line therapy**	**Second-line therapy**	**Third-line therapy**
N		89	53	34	14
Patients with any radiotherapy	N	16	7	3	3
	%	18,0%	13,2%	8,8%	21,4%
Incidence of radiotherapy (per patient with any radiotherapy per month ^(1)^)	Mean	0,06	0,17	0,07	0,17
	95% CI	0,04-0,08	0,09-0,25	0,05-0,1	0,-0,4
Total radiotherapy cost per patient with any radiotherapy (€ 2009)	Mean	2.814	2.814	2.814	2.814
Total radiotherapy cost per patient with any radiotherapy per month (€ 2009)	Mean	200	500	200	500
	95% CI	100-200	300-700	100-300	0-1.100
Total radiotherapy cost per patient (€ 2009)	Mean	506	372	248	603

^(1)^ month of follow-up.

### Transfusion

Transfusions were relatively rare, with 3.8% of all patients who received systemic therapy also receiving a transfusion. Consequently the mean cost for the generality of the sample is very low (€ 12).

### Surgery

24% of patients received surgery in combination with systemic therapy (Table 10). Surgery was more common in patients who had any response to systemic therapy (30.35%) as compared with those with no response (19,3%) (Tables 11 and 12). Surgery was among the most expensive categories of resource utilization, with a mean cost of € 7,390 per patient with any surgery. With reference to the generality of the sample, mean cost per patient with any response was equal to € 2,312, which is higher than the cost per patient with no response (€ 1.376).

**Table 10:** Summary statistics for surgery for patients receiving systemic therapy

		**Overall**	**First-line therapy**	**Second-line therapy**	**Third-line therapy**
N		208	147	112	41
Patients with any surgery	N	50	36	18	5
	%	24,0%	24,5%	16,1%	12,2%
Type of surgery					
Resection of primary tumor	%	9%	9%	0%	0%
Lymph node resection	%	21%	16%	3%	2%
All other visceral	%	22%	12%	7%	3%
Brain metastases	%	9%	5%	3%	1%
Isolated limb perfusion	%	0%	0%	0%	0%
Biopsy	%	12%	9%	2%	1%
Distant skin, subcutaneous or node	%	12%	9%	3%	0%
Lung	%	1%	1%	0%	0%
Total surgery cost per patient with any surgery (€ 2009)	Mean	7.390	6.368	5.670	7.638
Total surgery cost per patient (€ 2009)	Mean	1.776	1.560	911	931

**Table 11:** Summary statistics for surgery for patients with any response to systemic therapy

		**Overall**	**First-line therapy**	**Second-line therapy**	**Third-line therapy**
N		89	53	34	14
Patients with any surgery	N	27	13	8	2
	%	30,3%	24,5%	23,5%	14,3%
Type of surgery					
Resection of primary tumor	%	6%	6%	0%	0%
Lymph node resection	%	11%	7%	1%	1%
All other visceral	%	12%	5%	4%	0%
Brain metastases	%	5%	3%	1%	1%
Isolated limb perfusion	%	0%	0%	0%	0%
Biopsy	%	6%	3%	1%	0%
Distant skin, subcutaneous or node	%	5%	3%	1%	0%
Lung	%	1%	1%	0%	0%
Total surgery cost per patient with any surgery (€ 2009)	Mean	7.621	9.070	5.778	7.426
Total surgery cost per patient (€ 2009)	Mean	2.312	2.225	1.360	1.061

**Table 12:** Summary statistics for surgery for patients with no response to systemic therapy

		**Overall**	**First-line therapy**	**Second-line therapy**	**Third-line therapy**
N		119	94	78	27
Patients with any surgery	N	23	23	10	3
	%	19,3%	24,5%	12,8%	11,1%
Type of surgery					
Resection of primary tumor	%	3%	3%	0%	0%
Lymph node resection	%	10%	9%	2%	1%
All other visceral	%	10%	7%	3%	3%
Brain metastases	%	4%	2%	2%	0%
Isolated limb perfusion	%	0%	0%	0%	0%
Biopsy	%	6%	6%	1%	1%
Distant skin, subcutaneous or node	%	7%	6%	2%	0%
Lung	%	0%	0%	0%	0%
Total surgery cost per patient with any surgery (€ 2009)	Mean	7.119	4.841	5.583	7.780
Total surgery cost per patient (€ 2009)	Mean	1.376	1.185	716	864

## Conclusions

In the MELODY study data on resource use is collected based on patients stratification accordingly to treatment line, which implies that a given patient may be included in more than one line. This is the reason why in the present article costs per line are not examined, since the balancing cannot be found between the mean cost of the whole sample and the weighted mean cost of the strata. Instead, (Overall) costs are considered within two strata (patients with any/no response to systemic therapy) since the number of patients considered therein is stable within the different cost categories, so that the weighted mean cost of the two strata approximates the mean cost of the whole sample.

Moreover, it has to be noted that the reference period for calculating resource consumption by each patient corresponds to the follow-up period, which varies among patients. Therefore, the mean cost per patient is not directly referred to a standard time period (e.g. one year).

The following summary data must be appraised in the light of the above considerations, bearing in mind that the follow-up period is 17.5 months long on the average (Table 2) and that the balancing is rough between the mean cost of the whole sample and the weighted mean cost calculated on the two strata (any/no response) (Table 13).

**Table 13:** Summary costs per patient

	**% with any utilization**	**Mean cost per patient with non- zero utilization (€)**	**Overall cost per patient based on mean**^**(1)**^**(€)**	**Overall cost per responder based on mean**^**(1), (2)**^**(€)**	**Overall cost per non-responder based on mean**^**(1), (3)**^**(€)**
Hospitalization	9,8%	25.400	2.481	4.524	882
Hospice	5,6%	3.300	184	184^(4)^	184^(4)^
Emergency room	1,4%	300	4	4^(4)^	4^(4)^
Outpatient	40,5%	70	28	33	22
Radiotherapy	19,7%	2.814	555	506	591
Transfusion	3,8%	300	12	12^(4)^	12^(4)^
Surgery	24%	7.390	1.776	2.312	1.376
Total			5.0470	7.575	3.071

^(1)^ For the follow-up period (17,5 months on average). Patients with zero resource utilisation are included.

^(2)^ For patients with any response to systemic therapy.

^(3)^ For patients with no response to systemic therapy.

^(4)^ Overall data as a proxy.

The mean cost per patient for the generality of the sample is € 5,040. Hospitalisation is responsible for one half (49.2%) of it and surgery for more than one third (35.2%), so that both categories take up about 85% of the total amount. Radiotherapy is the third relevant category (10%). Of the remaining ones, only hospice is non negligible.

On the whole, these resources are supplied in a specialistic environment, for which hospitalization of patient is required. Only visits can be performed in outpatient setting.

The cost composition is emphasized in the former of the two strata considered (patients with any response to systemic therapy): hospitalization (59.7%) and surgery (30.5%) take up more than 90% of the cost for resources. Among patients with no response, instead, both categories together take up only 73.5%, where – on the other hand – hospitalization decreases to 28.7% but surgery increases to 44%; in this stratum the share for radiotherapy too is high (19.2%), when compared with the analogous in the former stratum (6.7%). Considering, moreover, that patients with any response cost on the average two and a half times compared to patients with no response (€ 7,575 vs € 3,071), one can infer that treatment profiles are remarkably different: in the former stratum hospitalization (where chemotherapy is administered) is prevailing, while in the latter surgery and radiotherapy come first.

Although the above mentioned limitations, this is the first study where the cost of treatment for a patient with advanced melanoma has been estimated in Italy. Even at the international level, few cost of illness studies can be found reporting such data. Some of such studies do analyse cost as a function of the illness stage; nevertheless, due to differences in methods, their results cannot be compared with the findings of the present study. Moreover, such studies are generally focused on the total cost charged to the national health system, from which they cannot derive a per patient cost based on of epidemiological information.

However, a study carried out in Spain reports cost data at patient level (referred to 2007) [21]. Based on a theoretical model, it concludes that higher costs are associated to patients with advanced melanoma. Only direct medical costs were considered, particularly hospitalization ones, broken down by four seriousness levels of the illness: detection, resection, surgical treatment of lymphatic spread, oncologic treatment of metastatic melanoma. As a first approximation, patients included in the fourth level might be considered homogeneous with those enrolled in the MELODY study. In the Spanish study two average per patient cost data (on yearly basis) are reported with reference to advanced melanoma: for patients with lymph node metastasis (€ 6,457) and for patients with visceral metastasis (€ 1,036). Size information of the two subset is not provided, so a weighted average cannot be calculated. But, assuming approximately equal sizes, an average value would result similar to that above reported for Italy (€ 3,456).

For the sake of completeness it is worthwhile reporting the results from three further studies, though no per patient cost data are there provided. In the first study, which is referred to France, the yearly (2004) cost is estimated for the French hospital system to treat patients with melanoma [22]. Such cost amounts to € 59 million, 27 (45%) of which are born for patients with metastasis. Main cost drivers are surgery (38%), follow-up evaluations (20%) and chemotherapy (17%). Authors conclude that costs for treating melanoma represent less than 1% of total French hospital system costs for cancer.

In the second and third study, the cost of melanoma was evaluated within a larger research focused on costs of all kinds of skin tumours. In particular, in the second study [23] cost data (2003) are reported relative to the hospital system in Germany, where about 20% of hospitalizations for skin tumours (62,384) are related to patients with melanoma (20,445), identified with ICD 10 code C43. For such patients, the total cost estimate vary depending on the resource evaluation method adopted: from € 59 million (evaluation with DRG tariffs) to € 55 million (evaluation with average cost per day stay). So, the average hospitalization cost per (C43) patient approximately ranges between € 2,900 and € 2,700.

In the third study cost data (2005) are reported for treating patients (here too identified with ICD 10 code C43) with skin tumours in Sweden [24]. The study, which estimated both direct and indirect costs, reports a total amount of € 142 million, of which direct medical costs represent 56%. Melanoma is associated to the highest financial burden (€ 80 million, of which 22 for direct costs). Dividing such total direct cost by the number of recorded treatment cases, an average cost per case is obtained of about € 2,000. Considering that for each patient more than one case on the average was recorded, also this data may be comparable with previously reported ones.

Before concluding, a recent review should be mentioned [25] where three cost-effectiveness studies and two cost-utility studies of chemotherapic treatment of metastatic melanoma were analysed. The authors conclude that the cost-effectiveness has not been widely demonstrated for treatment of metastatic melanoma and that a need exists for effective treatments that improve duration and quality of life.

As a conclusive remark, a message can be drawn from the present study: the cost for treating advanced melanoma is not particularly high (neither in Italy nor in other West European countries). In our opinion, this is mainly due to the fact that there are no effective treatmentsavailable, which can improve both duration and quality of life. Evidence of such opinion can be found in the low frequencies with which some resources are used, in particular hospitalization (less than 10%), considering that patients are hospitalized mainly for being administered an antitumoral therapy. Further evidence is provided by the above mentioned review [25], showing the poor cost-effectiveness of the analyzed treatments. Also the French study [22] confirms the low financial impact of the advanced melanoma treatment (less than 1% of total French hospital system costs for cancer). A medical need does therefore exist (as pointed out in most studies here considered) of more research and development investments in new effective and safe pharmacological treatments.

## Endnotes

^a^This is true for all the tables focused on response/non-response to therapy (i.e. tables regarding hospitalization, outpatient visits, radiotherapy, surgery), although it is most noticeable for hospitalization length of stay because this is an outcome that can be heavily influenced by a single patient with a long hospitalization. For example, if a patient had a very long hospitalization during first line therapy, and he did not respond to first-line therapy but did to a subsequent line of therapy, such hospitalization would be included in the Overall column in Table 4 (because the patient did respond to at least one line of therapy), and in the first-line column in Table 5 (because he did not respond to first-line therapy, which is when the hospitalization actually took place).

## Abbreviations

FDA: Food and Drug Administration; AJCC: American Joint Commission on Cancer; DRG: Diagnosis Related Groups; ATC: Anatomical Therapeutic Chemical; AE: Adverse events; ICD: International Classification of Diseases.

## Competing Interest

Dr. P. Ascierto is consultant for Bristol Myers Squibb, Merck Sharp and Dohme, Roche Genentch, was involved in Advisory Board for Bristol Myers Squibb, Merck Sharp and Dohme, Glaxo Smith Kline, Celgene, Amgen, Medimmune, Novartis, and has received honoraria from Bristol Myers Squibb, Merck Sharp and Dohme, Roche Genentch; MD A. Testori has received honoraria for participating to advisory boards to discuss treatment options in stage IV melanoma patients with pharm companies as BMS, Roche Amgen GSK Merk Celgene; Dr. P. Queirolo was involved in Advisory Board for BMS, Glaxo Smith, Roche Genetech.

## Authors’ contribution

All authors contributed to the design, analysis and interpretation of data; MM and CL were envolved in drafting the article. All authors revised the article and provided final approval.

## Aknowledgments

Research support: This study was sponsored by Bristol-Myers Squibb.
